# Invasion of privacy or structural violence? Harassment against women in public transport environments: A systematic review

**DOI:** 10.1371/journal.pone.0296830

**Published:** 2024-02-28

**Authors:** Sergio A. Useche, Natura Colomer, Francisco Alonso, Mireia Faus

**Affiliations:** INTRAS (Research Institute on Traffic and Road Safety), University of Valencia, Valencia, Spain; Walden University, UNITED STATES

## Abstract

**Background:**

Despite being neglected for several decades, and in many countries, public transport environments have transformed into a reflection of social disparities and inequalities. Among these issues, harassment–a pervasive and worldwide gendered dynamic–has been demonstrated to negatively impact women’s behavioral trends, daily opportunities, and health impacts, as well as safety and security outcomes.

**Aim:**

This systematic review aimed to examine a set of studies analyzing harassment against women in public transport environments, with a focus on key issues such as its prevalence, impact on transport dynamics, preventive strategies, and policing avenues documented in the scientific literature.

**Method:**

A total of 575 indexed articles were filtered using the PRISMA methodology, resulting in a final selection of 28 original articles directly addressing the issue up to December 2023. Search strategies were developed and implemented across WOS, Scopus, NCBI, Google Scholar, and APA databases.

**Results:**

Besides high frequency, widespread underreporting, and adverse effects on women’s safety, this review has identified correlations between harassment and travel behavioral adaptations. Furthermore, it reveals a noticeable disparity between the existing measures and those perceived as more effective by potential victims. These findings underscore the pressing need to listen to and promote the inclusion of women in decision-making regarding transport affairs.

**Conclusion:**

The findings of this systematic review suggest that, despite a slightly limited body of research, the impact of transport harassment on women’s health and welfare is consistently supported in the literature. In addition to being largely explained by existing inequalities rooted in social determinants, transit harassment further exacerbates gender gaps, gaining prospective importance for transport settings.

## Introduction

Whereas the increasing use of public transport for regular commuting seems essential nowadays for achieving transport sustainability goals [1–3], many empirical studies highlight that sexual harassment against women in transit environments is a fundamental global challenge to overcome [4–6].

However, despite the magnitude of this uncomfortable reality, many countries have not yet implemented preventive measures that contribute to increasing women’s perceived safety [7]. Indeed, some studies state that no effective gender policies or compelling specific measures have been implemented in most public transport systems and transit scenarios around the globe [8].

As a result, sexual harassment, apart from influencing women’s opportunities to use certain modes of transportation and limiting their ability to move freely [9, 10], contributes to structurally deepening existing gaps. For instance, females have been generally found to be more likely to use public transport for income-related status than men, as well as to simultaneously suffer victimizations, psychosocial impairments, and health threats in the transit environment [11].

### Is it gendered… or just “different”?

Although the literature on this issue has gained ground in recent years, key constraints such as the lack of consensus, cultural bias, and the absence of a comprehensive approach to recognizing what behaviors and events might be considered instances of transport harassment remain active. Furthermore, the existing studies measuring sexual harassment, apart from being really scarce in most countries, are often limited in terms of methodological rigor and operational clarity. This lack of advance in terms of scientific coverage makes it difficult to achieve a better understanding of the prevalence, features, and possible solutions for this issue, as well as to recognize a deep issue that remains, in most cases, simply underreported [12].

Overall, some criteria have been proposed for the recognition of sexual harassment, including the unwanted nature of the act, the sexual intention of the harasser, the existence of a pattern or repetition, behavior that is intrusive and of a sexual nature, and the generation of threat or fear [13]. Consequently, gender harassment typically includes crude, offensive, and derogatory sexual behaviors that reflect negative attitudes about girls and women in general, and specifically, “unwanted sexual attention” refers to uninvited, unwanted, and non-reciprocal sexual attention which is not welcomed by the recipient. Likewise, the unwanted nature of the encounter implies sexualization, intimidation, and discomfort [14].

Those unwanted sexual conducts comprise a wide range of behaviors that can be verbal (e.g., misogynistic comments, sexual gestures, insults, kissing noises, catcalling, whistles, unwanted conversational approaches -which might persist even after females’ rejection-, unfriendly and gendered comments -e.g., citing sexual names or graphic sexual comments about appearance-, other sexual demands or threats), nonverbal (e.g., staring, leering, sexual gestures, indecent exposure, picture taking, public masturbation), proximity (e.g., honking the horn, stalking or following someone), and/or physical (e.g., being too close, rubbing, touching, assault) [14, 15].

Furthermore, both harassment and other forms of violence against women in public spaces occur routinely around the world [16, 17], and these come as a result of innumerable cultural factors, social misconceptions, and historical practices, which have unjustifiably led to its *normalization* in many countries, suggesting a marked structural character. These heterogeneous factors are related to traditional gender roles that still exist in many societies and the ‘macho’ culture derived from this social phenomenon. For example, in societies where masculinity is narrowly defined and associated with dominance and control, harassment of women in public transportation can occur as a way for harassers to reaffirm their power and control over women in a shared space.

### Harassment-related dynamics and structural determinants

Although still low number in number, ecological frameworks have been applied to studies on violence against women, overall suggesting that there are factors exogenous to individual women that interact to increase their vulnerability to violence [18]. Among their findings, the multidimensional relationships between the social determinants of health (e.g., transportation resources, socioeconomic status, housing location, neighborhood safety) and interpersonal violence against women, including harassment, stand out.

From a structural approach, women’s harassment experiences are usually approached because of their intersections among micro- (relationships in the immediate context), meso- (links between individual and other ambits of involvement, such as family, networks, workplace, community, etc.), and macro-levels (social institutions and structures) as factors producing and reproducing violence among women [19]. Indeed, recent studies state gender as a constitutive element of social and transport dynamics, as it organizes social life in hierarchical, mutually exclusive categories, thus contributing to maintaining subordinate positions, whether material or ideological, among people within families, households, or communities [19, 20].

In parallel, the characteristics of different urban environments may influence the prevalence of these types of behaviors [21]. In this sense, public transport and its facilities are spatially prone to harassment situations towards women [22]. Public transportation is often considered spatially prone to harassment due to various interrelated reasons. Firstly, the high passenger density in confined spaces creates a conducive environment for unwanted behaviors, as physical proximity can increase the opportunity for harassment. The lack of privacy in these shared spaces can make victims feel more vulnerable and less likely to report incidents. Additionally, the hierarchical structure and design of some public transportation systems can facilitate impunity, allowing harassers to easily blend into the crowd and escape identification. This was highlighted in the last report by The United Nations Women on harassment, depicting that, along with the job-related sphere, transport is a key scenario in explaining and eradicating harassment against women [23].

### Fear of harassment in public transport: Psychosocial, health and behavioral implications

The most commonly documented psychosocial implication of transport harassment is the so-called fear of violence, which leads to gender-based differences in behavior [24], as well as its spatial manifestation of gender-based power relations [25]. For instance, this fear leads women to use gender-specific precautions, such as avoiding places at night and relying on escorts for protection. However, such behavioral adaptations have many negative consequences in women’s daily lives, as well as in their subjective travel experience [24]. Some specific examples of the negative consequences of women having to take alternative routes or avoid certain places at night include the potential increase in commuting time or the economic costs of transportation. This, in turn, can impact the efficiency and comfort of their journeys. Nevertheless, due to their high frequency and, sometimes, cultural tolerance, social constraints tend to undervalue or normalize these occurrences. Even worse, they may contribute to assuming them as part of daily life in the transportation sphere [26].

Bearing in mind these circumstances, psychological consequences mediated by fear and concern after harassment could lead to underestimation, under-reporting, and lack of action. For instance, in the New York and London transport systems, the normalization of sexual harassment has been suggested as a reason for the under-reporting of unwanted sexual behaviors [27–29]. More commonly cited reasons for not reporting unwanted sexual behavior on public transport by victims and bystanders include understanding the incident as not being serious enough to report and not knowing how to or being unable/unwilling to report it. It has also been argued that wider prejudices keep the problem hidden, with women downplaying incidents because the emphasis is often on the victim rather than the perpetrator’s behavior (e.g., a female traveling alone in an empty carriage late at night may feel at fault because of their choice of travel time [7]).

Contextually, harassment can be developed in any circumstance in the traffic environment, and this violence against women is more common when the transport vehicle or carriage is either crowded (overcrowding may allow perpetrators to carry out their actions unnoticed and without consequence) or completely empty [30, 31]. Consequently, behavioral studies have documented how most women try to avoid facing environments they may consider unsafe, causing them to change their commuting routines regarding the time chosen for journeys, the route followed, or the transport means used [32]. Once more, the expression of fear of sexual harassment or assault and concern for their own safety is constructed as an integrated gender response. Indeed, some studies have documented how, far from punishing street offenders, women are often socially blamed for not embracing risk avoidance measures, such as constraining their mobility patterns, changing their appearance or lifestyle, or just avoiding traveling alone [24].

### The current study

Bearing in mind the aforementioned considerations, the present study conducts a systematic review of women’s perception of sexual harassment in public transport, as well as its impact on their transport dynamics through research conducted worldwide. The following issues have been specifically considered:

1. The frequency of harassment against women reported on public transportation scenarios and the specific conditions and circumstances under which it tends to take place.
2. The impact of harassment on women’s perception of public transportation, their perception of safety, and its repercussion on their transportation dynamics.
3. The strategies carried out to reduce the chances of being assaulted or harassed on public transport.
4. The measures perceived as most effective in reducing harassment in transit environments.

This systematic review seeks to provide information on the perception of sexual harassment in public transportation from the perspective of women, examining its impact on their commuting dynamics and offering a global view of the phenomenon. By addressing this issue on an international scale, it contributes to a broader understanding of the conditions and circumstances of harassment, providing a solid foundation for the formulation of policies and prevention strategies.

## Methods and materials

### Methodological approach

Systematic reviews involve mapping the literature with a transparent and systematic methodology to find and explain a research question in detail, by means of literature-based and fixed criteria allowing researchers to reach reliable conclusions on the state of affairs of literature on a certain topic, or a set of them. Therefore, it involves a process of careful researching for high-quality studies to assess their quality and synthesize their main results and findings [33].

To achieve this, we used the Arksey and O’Malley (2005) [34] methodology to propose recommendations that clarify and enhance each stage of the framework. The five stages typically followed are described below:

1. Identifying the Research Question.
2. Finding Relevant Studies.
3. Selecting the Studies.
4. Charting and Collating the Data.
5. Summarizing and Reporting the Results.

### Step 1: Identifying the research question

As previously stated, the aim of this systematic review is to identify the number and type of studies investigating harassment on public transport. In this sense, we seek to know the prevalence of this type of harassment in different parts of the world, the prevention strategies used by women, its impact on their psychosocial health, transport dynamics, and behavioral adaptation measures. Hence, and with the aim of highlighting possible discrepancies (and concordances) in the results, the results include a summary and a topic analysis of all the chosen articles.

### Step 2: Finding relevant studies

The present research was carried out following the PRISMA guidelines for the notification of systematic reviews [35] (see S1 Checklist for further information). The databases used for the preliminary literature search were the Web of Science, the American Psychological Association (APA), Scopus, the National Center for Biotechnology Information (NCBI), and Google Scholar. Other lists of references of different scope reviews in primary research, potentially eligible and not captured by our search strategies, were also reviewed.

The search contained literature published from the beginning of the database and included the first week of December 2023. The terms searched for included: “public transport”, “harassment”, “gender differences”, “risk perceived”, “security” “experiences”, “affectation”, “impact”, “women”, “female”, “bus”, “train" and other similar words. These terms were identified after a review of the titles and keywords of the articles we found during our preliminary search. The whole set of terms appended in the search strategy is presented in Table 1.

**Table 1:** Search strategy, including key index terms, Boolean operators, and successive search steps.

#1	((transit environment [All Fields] OR public transportation [All Fields] OR public transport [All Fields] OR commuters [All Fields] OR train [All Fields] OR bus [All Fields] OR metro [All Fields] OR subway [All Fields] OR taxi [All Fields] OR cab [All Fields]) AND (women [All Fields] OR female [All Fields] OR girl [All Fields] OR gender [All Fields]) AND (Harassment [All Fields] OR harasser [All Fields] OR risk perceived [All Fields] OR security [All Fields]))
#2	((gender differences [All Fields] OR experiences [All Fields] OR testimony [All Fields] OR testimonials [All Fields] OR psychosocial impact [All Fields] OR health outcomes [All Fields] OR affectation [All Fields] OR impact [All Fields] OR fear of crime [All Fields] OR fear of victimization [All Fields] OR fear of harassment [All Fields] AND (transport [All Fields]) AND (measures [All Fields] OR policy [All Fields] OR control [All Fields] OR preventive [All Fields] OR prevention [All Fields]))
#3	#1 AND #2

### Step 3: Selecting the studies

Articles that did not refer to our research objective were excluded during this stage. Additionally, articles on street harassment were excluded if they did not focus on public transportation. Publications in the form of letters, conferences/summaries, editorials, case reports, protocols, or case series were not selected. We also restricted our eligibility criteria to articles published in English and Spanish, publicly available or possibly requestable from the library system used.

All authors initially and independently assessed a subset of titles and summaries and then met up in order to discuss and solve any discrepancies in regard to both the search outcomes and filtered studies.

### Step 4: Charting the data

The articles that fitted the inclusion criteria were critically reviewed using the descriptive-analytic method [34]. For each eligible article included, the following data were extracted and registered: title of the article, author(s), year of publication, country of the study, study design (including sample size), method, main findings, and highlight results.

### Step 5: Collating, summarizing, and reporting the results

The graphed data were summarized in tables, followed by the descriptive data, analyzed through a thematic-based organization strategy. For this purpose, the main sections and issues used for empirical research were summarized in successive columns (see Table 1). Once their relevant characteristics and main findings were summarized, the quality of the articles included in the systematic review process was assessed using the Critical Appraisal Skills Program (CASP) tool, whose main utility is to perform a quality assessment of the studies analyzed, to ensure that the results are not significantly altered or biased by possible technical deficiencies present in these sources.

Additionally, a network analysis will be presented using a thematic-based successive strategy for categorizing studies aimed at exploring the relationships among core study features in this field (please refer to Fig 3). Finally, with the aim of comparing the specific issues and trends addressed by investigations in this field over the last few years, a discursive analysis of the analyzed research outcomes has been conducted using the VOS viewer tool. Among many other features, this tool enables the construction and visualization of bibliometric networks discriminating by their years of publication (see Fig 4).

### Ethics

The protocol for this systematic review study was evaluated and approved by the Ethics Committee of the University Research Institute on Traffic and Road Safety (INTRAS) at the University of Valencia, certifying that it responded to the general ethical principles applicable to this type of research (IRB approval number RE002181122).

## Results

### Search results

**Fig 1:**
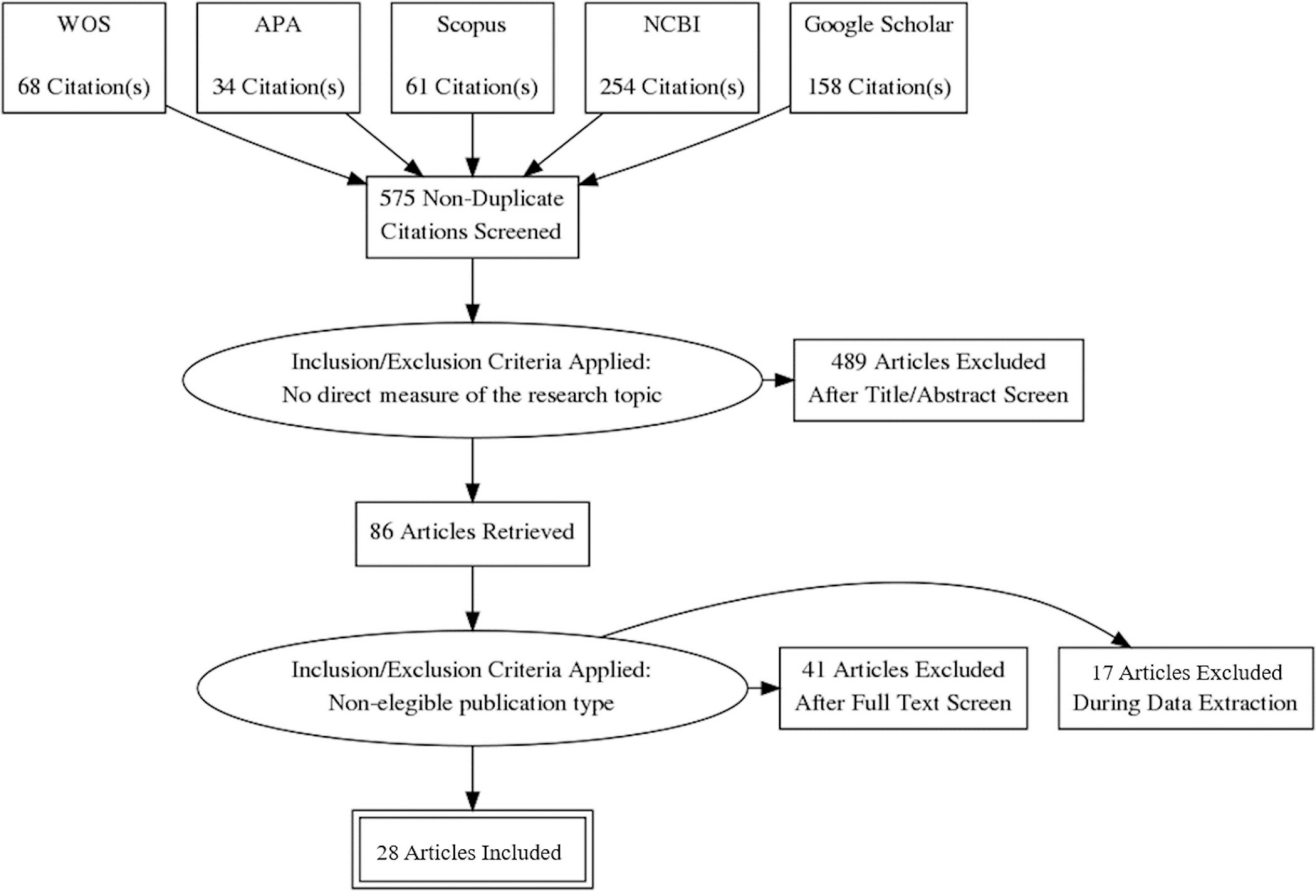
PRISMA flowchart for this systematic review. *Abbreviations: WOS (Web of Science); APA (American Psychological Association); NCBI (National Center for Biotechnology Information).

The searched words identified a total number of 575 possible articles to be analyzed (duplicated or non-accessible elements were ruled out). A subsequent manual selection of the papers that adjusted to the core aim of the present systematic review, as well as to the selection criteria was performed, leaving as a result 2 discards and 28 eligible articles to be handled. Further, the data source searching strategy and step-by-step selection process of the preliminary and filtered research outcomes are available in Fig 1.

### Characteristics of eligible research articles

**Fig 2:**
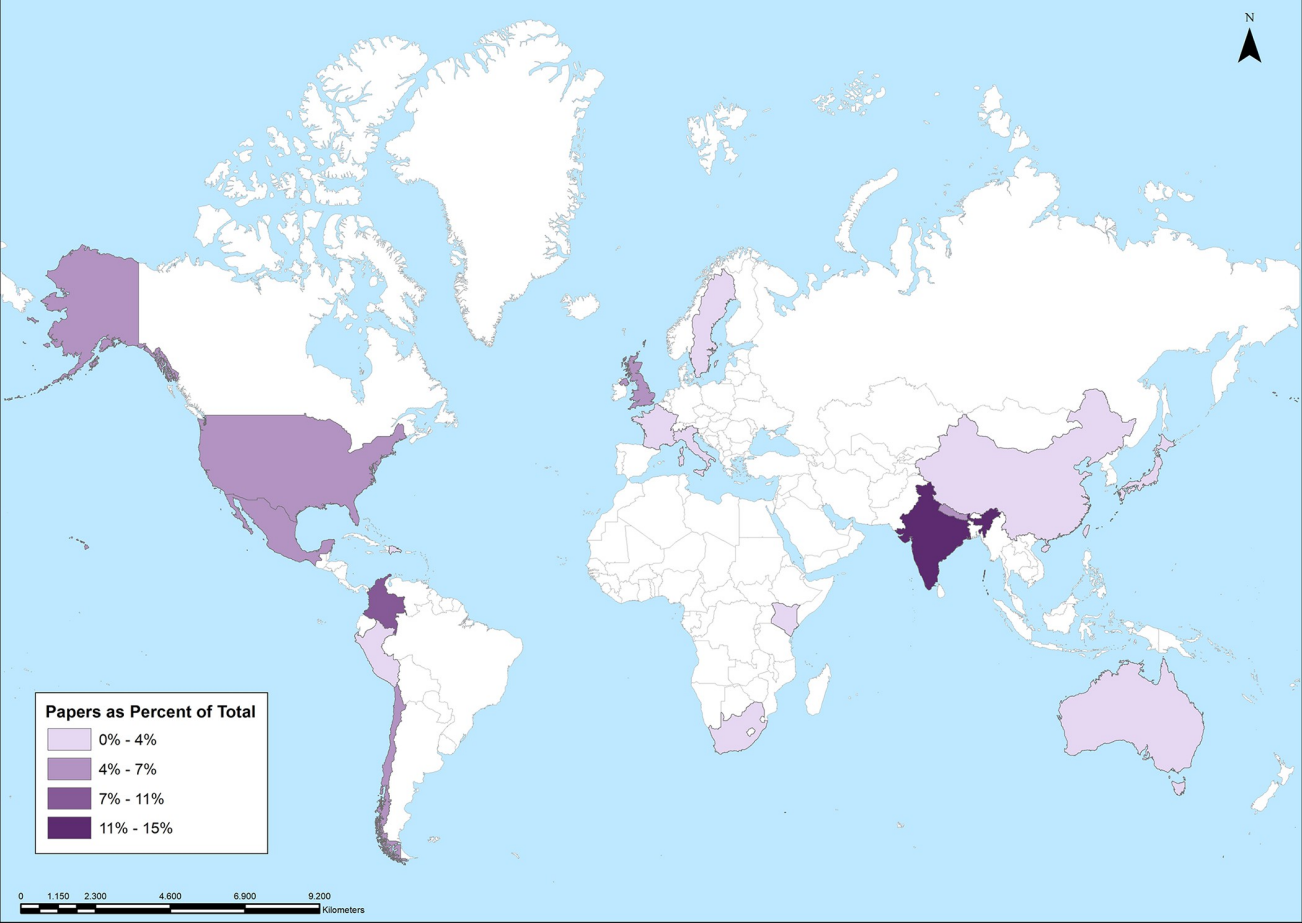
Geographical distribution (country of origin) of the studies covered by the systematic review process meeting the inclusion criteria. *Source*: Own elaboration.

Moreover, the studies were conducted in 17 different countries: India (n = 4), Colombia (n = 3), The United States (n = 2), Nepal (n = 2), The United Kingdom (n = 2), Mexico (n = 2), Chile (n = 2), France (n = 1), Peru (n = 1), South Africa (n = 1), Sweden (n = 1), Kenya (n = 1), The Dominican Republic (n = 1), Japan (n = 1), China (n = 1), Australia (n = 1) and Italy (n = 1) (see Fig 2). In addition, one article that studies samples from 18 different cities on five continents was found. Overall, four continents are represented, depicting the prevalence of harassment on public transport as a global issue, regardless of socioeconomic conditions.

The methodology followed by these studies was cross-sectional, usually using online surveys or face-to-face interviews (n = 28). In turn, there are studies with quantitative (n = 18), qualitative (n = 7), and mixed (n = 3) analysis. Quantitative analysis provides great value because of the sample sizes it is capable of analyzing and the statistical tests that can be performed with the bulk of the data. This leads to obtaining solid and repeatable results. Similarly, qualitative analysis provides valuable information because it can explore and reach a great depth of information in its responses when its focus is on a few subjects. Although generally more time-consuming, research using a mixed approach can be very enriching by combining the advantages of both types of methodology.

The selected articles cover at least one of the objectives suggested in this systematic review. In this sense, 12 articles evaluated the frequency and/or prevalence of harassment on public transport, concluding that most women had been subjected to some type of inappropriate behavior during their lifetime. The different types of harassment experienced in public transport facilities, whether these were vehicles, stations, or stops, are also detailed.

Moreover, a considerably large number of investigations address specific changes in women’s transport dynamics and their sense of safety (n = 12). The general conclusion is that women do not travel freely because of the insecurity caused by either having suffered an episode of harassment or the possibility of future harassment. Prevention strategies employed by female travelers were reported in seven (i.e., one-fourth) of the 28 articles analyzed. Overall, these sources also delve into behavioral adaptations, such as changing means of transport and avoiding specific areas and times considered unsafe by women. Additionally, instances of sexual harassment are typically described in crowded and/or empty scenarios of public transport stations, vehicles, or the pathways to and from them.

Finally, four of the analyzed investigations delve into describing the measures taken by different types of entities or government bodies that are perceived as most effective, including security cameras, police patrols, and women-only transportation. In addition, some of the articles sporadically reference other issues, such as the personal consequences for victims of harassment or sexual violence in public transport, or the characteristics that make public transport a conducive space for harassers. The full set of appended (and analyzed) studies in this systematic review is presented in Table 2. The table displays (from left to right) the different key features and conclusions from these empirical sources following a structured format.

**Table 2:** General characteristics of the eligible studies.

Author(s), publication year	Country	Study aim(s) and setting	Method	Results (main outcomes)	Key limitations
Loukaitou-Sideris & Fink, 2009 [36]	The United States	To assess women’s perception of safety in traffic, using a survey.	Quantitative and Cross-sectional (n = 131)	Women have different safety and security needs, but most do not think that organizations should use specific programs to deal with them. Differences are demonstrated between the security needs of female passengers and the tactics used by the relevant authorities.	(1) Self-report^1^
Ceccato et al. 2021 [37]	Sweden	To analyze the impact of fear on women’s mobility and precautionary behavior, ain addition to the situational characteristics of their daily commute.	Quantitative and Cross-sectional (n = 1122)	Greater cautionary behavior on trains and subways is associated with prior experience as a victim of sexual harassment. Furthermore, young women tend to avoid particular stations or routes at certain times, particularly when there is poor signage.	(1) Self-report^1^ (2) Not representative (3) Not generalizable
Lea et al., 2017 [20]	India	To identify the strategies women take to manage the threat of harassment, through accounts of assaults collected on a crowdsourcing platform.	Qualitative and Cross-sectional (n = 137)	The three most common strategies are remaining silent, running away from the situation and resisting the assault.	(1) Self-report^1^ (3) Not generalizable (4) Small sample
Orozco-Fontalvo et al., 2019 [38]	Colombia	To assess the factors affecting women’s perceived risk of sexual harassment when using public transportation.	Quantitative and Cross-sectional (n = 500)	Almost three quarters of participants have been sexually harassed when using public transport. The number of people in the vehicle, nighttime hours, and poor lighting are all thought to raise the perceived risk of female users.	(1) Self-report^1^
Mishra & Lamichhane, 2018 [39]	Nepal	To determine the prevalence of and factors leading to sexual harassment on public transport among female students on a university campus, using a self-administered questionnaire.	Quantitative and Cross-sectional (n = 396)	80% of the women surveyed said they had suffered some form of harassment, with physical, verbal and non-verbal harassment being the most common. The most typical responses were to reprimand the harasser, keep quiet, or get off at the next bus station.	(1) Self-report^1^ (2) Not representative (3) Not generalizable
Lewis et al., 2021 [40]	The United Kingdom	To present women’s real experiences of sexual harassment on the subway, using semi-structured interviews.	Qualitative and Cross-sectional (n = 29)	The frenetic pace of the subway, the lack of responsive of women, and civilian disinterest all contributed to harassment.	(1) Self-report^1^ (4) Small sample
Quinones, 2020 [41]	Colombia	To address women’s experience of sexual harassment in public spaces, especially on public transport (vehicles, stations, stops and walking trips), using an online questionnaire and semi-structured interviews.	Mixed and Cross-sectional (n = 1,338)	The prevalence of sexual harassment on public transport varies by age group and social class. However, due to the perception that accusations are pointless, very few are made.	(1) Self-report^1^
Neupane & Chesney-Lind, 2014 [42]	Nepal	To examine the sexual harassment that university women have experienced, concentrating on the severity, frequency, and type of harassment.	Mixed and Cross-sectional (n = 238)	None of the participants reported any of these assaults to authorities, despite the severity of some of them. Public transportation encourages harassment because it gives males opportunities and anonymity while posing little risk of social or legal repercussions.	(1) Self-report^1^ (2) Not representative (3) Not generalizable
Tripathi et al., 2017 [43]	India	To present the incidents of sexual harassment experienced and witnessed, as well as the risk perceptions of a group of female students in higher education.	Quantitative and Cross-sectional (n = 200)	Sexual harassment situations largely take place on buses. According to the study, sexual harassment is widespread in transportation, and is rarely noticed by other users.	(1) Self-report^1^ (2) Not representative (3) Not generalizable
Madan & Nalla, 2016 [44]	India	To analyze differences in perceptions of sexual harassment in public spaces and its perceived severity as a result of gender, using a questionnaire.	Quantitative and Cross-sectional (n = 1,389)	In most forms of public transportation, the perceived likelihood of sexual harassment correlates with actual self-reported victimization.	(1) Self-report^1^
Alonso et al., 2020 [45]	The Dominican Republic	To evaluate the potential relationships between perceived safety (in urban scenarios and public transportation settings) and daily travel-related behaviors and patterns through a national survey.	Quantitative and Cross-sectional (n = 1,026)	The variables of age, city size, education, and perception of safety in the urban environment influence the choice of transportation, in additional to the respondents’ experience as victims.	(1) Self-report^1^
Infante-Vargas & Boyer, 2021 [46]	Mexico	To analyze the violence faced by women in their daily lives, re-victimization and the psychological effects of these incidents, through a survey and an interview.	Mixed and Cross-sectional (n = 611)	After the event itself, women may experience a variety of effects, such as restrictions on their mobility, alongside financial and emotional repercussions.	(1) Self-report^1^
Coppola & Silvestri, 2020 [47]	Italy	To examine the security perceived by travelers at railroad stations, through a methodology based on Probit and Logit ordered choice models.	Quantitative and Cross-sectional (n = 240)	The variables that have the most influence on how stations are perceived as secure are assaults, harassment, and theft.	(1) Self-report^1^ (4) Small sample
Shibata, 2020 [48]	Japan	To analyze the issue of women being groped on public transportation and its impact on the victims.	Quantitative and Cross-sectional (n = 400)	Twenty-five percent of the participants have been groped more often on trains than buses. Although women-only carriages are thought to be an adequate solution, other measures, like surveillance cameras or increased police presence are believed to be more effective.	(1) Self-report^1^
Soto et al., 2022 [49]	Colombia	To describe the relationships between sociodemographic characteristics, travel situations, system design features, and fear of crime on public transport.	Quantitative and Cross-sectional (n = 500)	Fear of crime and perception of safety on public transport are significantly correlated, especially among women.	(3) Not generalizable (5) Data limitations
Vasquez-Henriquez et al., 2020 [50]	Chile	To evaluate travel experiences on Twitter, focusing on gender-related differences. The research analyzed more than 400,000 tweets addressing the topic of transportation.	Quantitative and Cross-sectional	Men struggle to articulate thei emotions, whereas women write about both their positive and negative emotions regarding travel. The biggest gender disparity in the transportation sector is harassment.	(5) Data limitations
Ceccato and Loukaitou-Sideris, 2022 [51]	Worldwide	To assess changes in perceptions of safety in traffic environments, through a survey administered to university students across six continents.	Quantitative and Cross-sectional (n = 13,323)	Avoidance strategies that deterred some passengers from traveling at particular times, by routes and in travel settings that are considered particularly unsafe, or that caused them to completely avoid using public transportation, all of which had an impact on student mobility.	(1) Self-report^1^
Sagaris & Tiznado-Aitken, 2020 [52]	Chile	To assess the implications of equality on women’s travel patterns and sustainable transport.	Quantitative and Cross-sectional	Women’s mobility is restricted by obstacles related to unsafe public transport environments. Access to transportation is unequal, with women walking significantly more than men.	(1) Self-report^1^ (2) Not representative (3) Not generalizable
Lebugle, 2017 [53]	France	To assess the experience of interpersonal violence in different living spaces, using the VIRAGE survey and a representative sample of people.	Quantitative and Cross-sectional (n = 27,268)	Different profiles were established: 1) insults without violent action; 2) catcalling; 3) physical violence; 4) sexual incidents; 5) sexual violence.	(1) Self-report^1^
Dunckel, 2016 [54]	Mexico	To analyze how women deal with violence and harassment on public transport, through testimonies, discussion forums and surveys.	Qualitative and Cross-sectional (n = 35)	The data shows that gender-based violence in the public transport system restricts women’s mobility and accentuates the gender gap.	(1) Self-report^1^ (3) Not generalizable
Wa & Samper, 2006 [55]	Kenya	To present women’s testimonies of abuse, robbery, kidnapping, rape and sexual harassment.	Qualitative and Cross-sectional	The article proposes strategies to tackle the issues and personal risks related to matatu, expressing a powerful comment on life.	(1) Self-report^1^ (3) Not generalizable
Lynch & Atkins, 1988 [56]	The United Kingdom	To analyze how women’s fears regarding harassment and assault influence their use of transportation, using a survey.	Quantitative and Cross-sectional	High levels of perceived insecurity were found, particularly when it came to walking at night, in parks, subways, and while waiting for public transportation in remote locations.	(1) Self-report^1^ (2) Not representative
Reed et al., 2019 [57]	The United States	To analyze the prevalence of sexual harassment among US adolescent girls, which was divided by type, place of occurrence and perpetrators, its association with substance use and poor mental health.	Quantitative and Cross-sectional (n = 159)	In the six months prior, 65.4% of the surveyed adolescents experienced sexual harassment events in public spaces (including public transport environments, schools and neighborhoods). Also, having witnessed or suffered these events is associated with substance abuse and poorer mental health outcomes.	(1) Self-report^1^ (2) Not representative (3) Not generalizable (4) Small sample
Gopal & Shin, 2019 [58]	India	To expose the impact of rail transport on women’s lives in a developing world country.	Qualitative and Cross-sectional (n = 51)	Due to the safety precautions put in place, women largely have a positive travel experience on the Delhi Metro. Nevertheless, they still encounter harassment in the metro spaces, so they develop behavioral strategies to avoid risky situations.	(1) Self-report^1^ (2) Not representative (3) Not generalizable (4) Small sample
Vanderschuren et al., 2019 [59]	South Africa	To analyze the different mobility patterns of women and men.	Quantitative and Cross-sectional	Women are subjected to harassment more frequently, which influences their choice of transportation.	(1) Self-report^1^
Medina & Zapana, 2016 [60]	Peru	To expose women’s experiences, attitudes and views about sexual harassment in the street.	Qualitative and Cross-sectional	Young women believe that sexual harassment in the street is a visible problem, and that verbal, expressive and physical manifestations of abuse in these settings are considered threatening.	(1) Self-report^1^
Ison et al., 2023 [61]	Australia	Identify experiences of sexual violence and harassment in public transportation.	Qualitative and Cross-sectional (n = 41)	The participants, in sharing experiences of violence on public transportation, also described several daily situations that influenced the way (i.e., day, time, transport means) they traveled. It is argued that addressing this problem from a functional approach requires a primary prevention focus on public transportation.	(1) Self-report^1^ (4) Small sample
Lee et al. 2024 [62]	China	Analyzes users’ acceptance of driverless public buses and ridesharing services and how individual characteristics affect their perceived risks of crime occurrence and victimization.	Quantitative and Cross-sectional (N = 1,090)	43% of the participants responded that the crime possibility is high, 39% stated they do not want to use driverless public buses or ridesharing vehicles, and 30% think the possibility of being a victim is high.	(1) Self-report^1^

Notes: ^1^The limitation of using self-report instruments refers strictly to the possible response biases derived from them and not to their value as measurement tools.

### Network analysis

In a subsequent analysis phase, as mentioned before, a network analysis was performed. This procedure was conducted to explore potentially useful data and relationships among studies, examining which topics have been addressed by them, where they have been performed, and what study designs and methods are principally employed to cover the topic in applied research.

**Fig 3:**
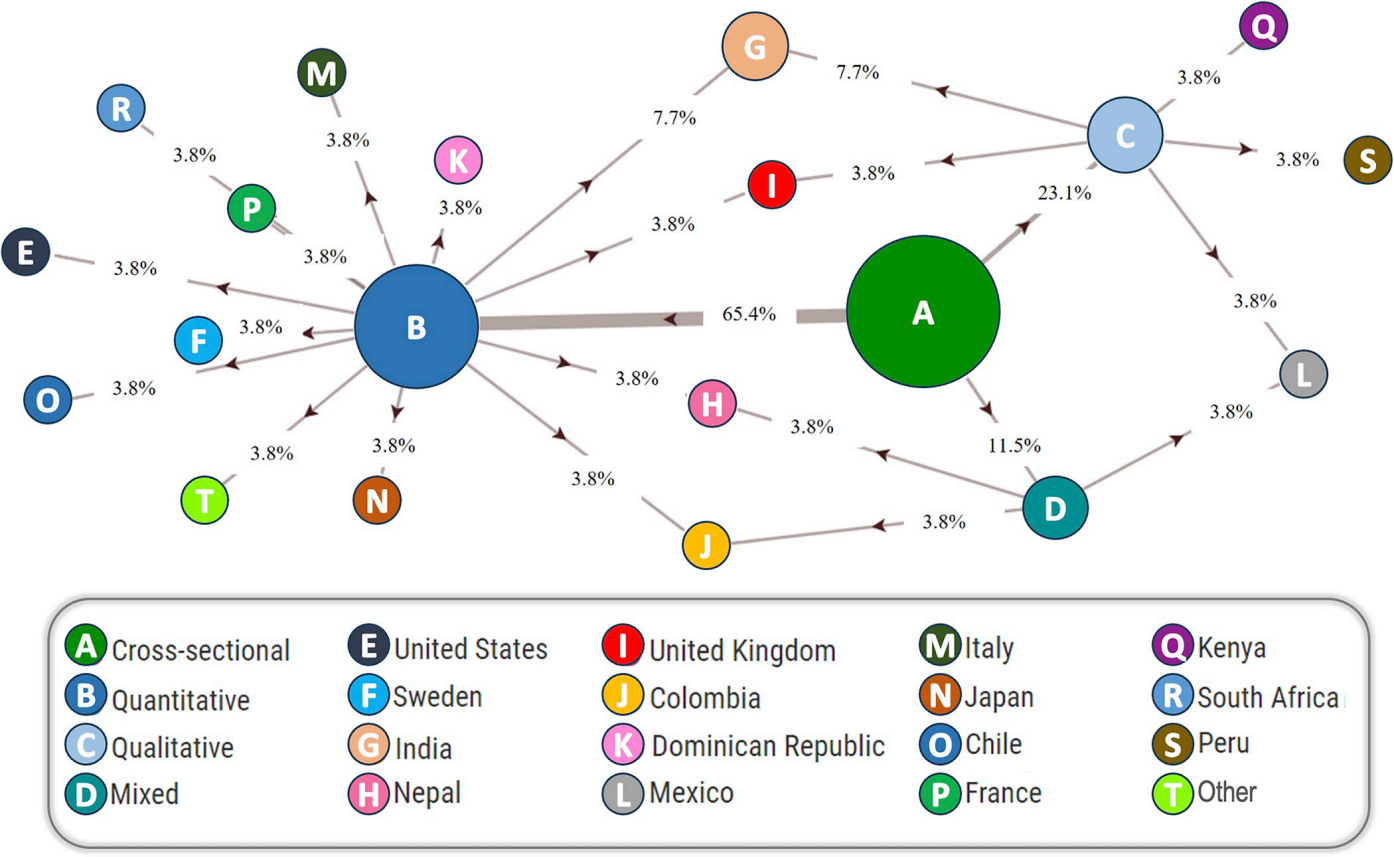
Node relationships among the analyzed studies addressing women’s harassment in transit environments. Numbers over/below vectors indicate the number of studies addressing the highlighted issues simultaneously.

The network relationships among the core features of studies addressing harassment issues in transport are shown in Fig 3, in consideration of three factors: study typology, methodology, and country of study. Overall, the obtained network relationships help depict the existence of key methodological commonalities among the studies included in the systematic review process after applying the selection filters and analyzing their specific contents.

Principally, observational/cross-sectional studies are the most used for addressing women’s harassment issues, and 65.4% of them comprised quantitative analysis approaches. On the other hand, 23.1% followed predominantly qualitative data analytic practices and 11.5% mixed-method approaches.

Furthermore, and related to the notorious lack of research in most countries (i.e., in most countries, only one study was conducted), a few geographical and conceptual connections were found among them. However, the case of India is slightly different, as the country offers both quantitative and qualitative studies on women’s harassment in transport, even though no mixed-method study has been targeted there.

Finally, it stands out that all the mixed-method studies were performed in emerging countries (i.e., Colombia, Mexico, and Nepal), while this type of research approach was not found in studies performed in high-income countries.

**Fig 4:**
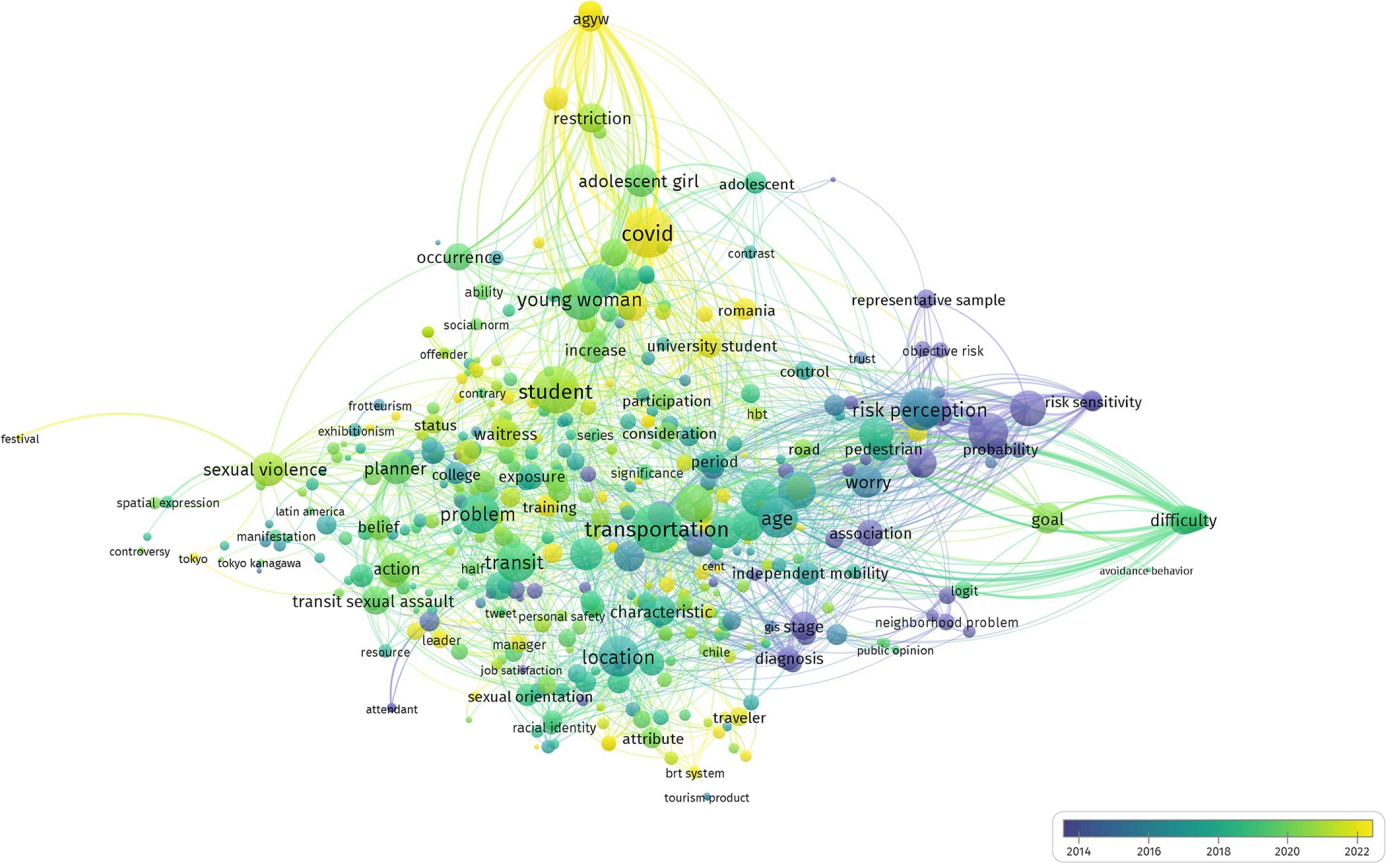
Analysis of the discourse trends of the selected articles according to the year of publication of the handled research papers.

Regarding the content of the articles, an analysis of the word communities most present in the text body has been conducted to extract discourse trends. This analysis allows examining relationships in terms of identifying key terms present in the discourse. Additionally, and concerning the temporal aspect (i.e., specific research trends over time), the analysis has segmented the contents of the papers into different year-based periods, assigning different colors to terms or relationships based on the temporal period in which they appear with greater frequency or relevance, as shown in Fig 4.

The graphical results depict a marked and successive change in research trends between the years 2014 and 2022. Specifically, while most of the terms and topics researched in earlier years -especially between 2014 and 2017- corresponded with greater frequency, albeit not exclusively, to risk-related factors (e.g., perception sensitivity, worry, trust), research trends in the period 2017–2019 seem to have emphasized topics related to age, places, transport problems, and specific victim profiles with greater strength. On the other hand, more recent research (2020–2023) has shown to address topics such as sexual violence, offender-related profiles and behaviors, and, of course, the impact of COVID-19 dynamics on women’s travel behavior, fear of crime, and sexual violence-linked outcomes, as well as practical actions and policy planning in these regards.

### Evaluation of the quality of the selected studies

**Fig 5:**
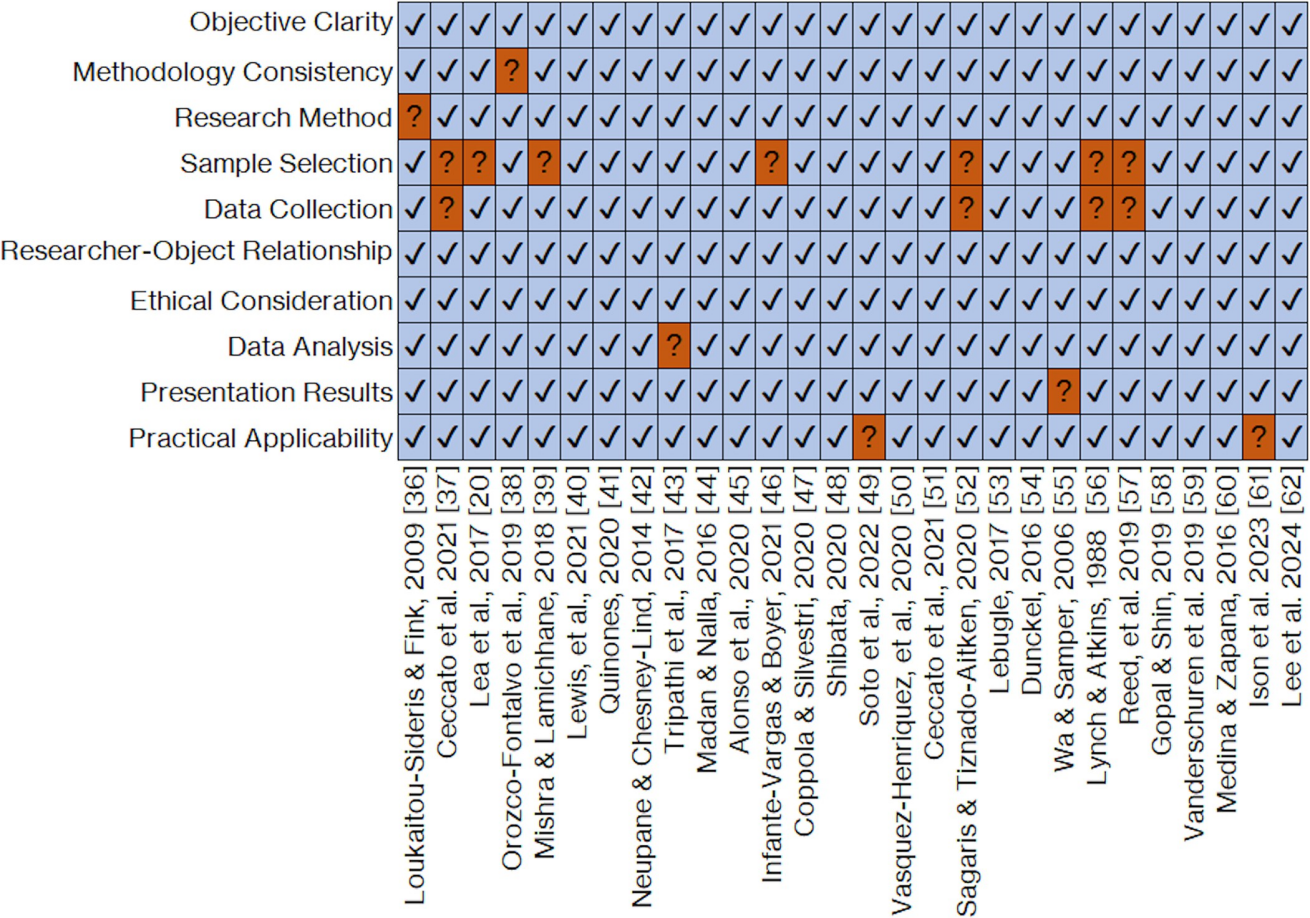
Item-by-item evaluation of the quality of the selected articles using the "Critical Appraisal Skills Programme" approach.

In order to ensure that no selected study could interfere with or distort the conclusions of this systematic review, the Critical Appraisal Skills Programme (CASP) quality assessment tool was applied. This methodology makes it possible to assess the level of rigor, credibility, and relevance of a study by means of ten questions focused on the key quality-related aspects of applied scientific research, guaranteeing reliability, validity, and methodological rigor [63]. According to our CASP-based analysis results, whose detailed features are presented in the vertical columns of Fig 5, all of the selected studies can be understood as having a low risk of bias and acceptable levels of overall quality. This endorses the decision to include them for analytic purposes in the present systematic review.

## Discussion

This systematic review aims to examine the number and type of studies that have analyzed harassment against women in transport environments, as well as their core psychosocial impacts, commonalities, and potentially stated implications for policy.

Concisely, a significant number of studies addressing this issue and their outcomes conclude that being a woman is indeed the most relevant risk factor leading to harassment in this context. However, this was not the aim of this study; instead, it served to highlight the importance of properly documenting this phenomenon. It addresses the needs that could contribute to developing further knowledge, measures, and strategies to protect women in transit environments. Therefore, the actual discussion of this paper can be found from now on.

### Is this a cross-cultural issue?

The studies included in our review tended to underline that cultural norms (entrenched in male violence, male dominance, social stigma, and cultural norms of patriarchy) and institutions maintain social constructions of gender around the world. Therefore, some recent studies have stated that sexual harassment in public areas can be understood as a manifestation of a latent *sexual power imbalance* endorsing implicit control mechanisms over public space, their female users, and a subsequent increase of abuse-related behaviors, as a result of the intersection between gender, social roles and, frequently, individuals’ age [26, 64, 65]. At this juncture, research conducted in multiple and culturally different countries exposes the high prevalence of physical, verbal, and (most frequently documented) sexual harassment in public transport [6, 43, 53, 66].

At the statistical level, and according to Chokalingam and Vijaya’s (2008) [67] research on 100 women in India, nearly two-thirds (63%) of the victims reported being subjected to multiple forms of a particular form of sexual harassment known as "eve teasing" (a term used in some parts of Asia to describe the harassment of women in public places). Another study reported that 39% of females surveyed in Jakarta, Indonesia, reported being sexually harassed in public transport and also noted that such harassment happened to them frequently [68].

This specific type of victimization occurs even in high-income economies and/or highly developed countries (that are not the same), such as North European and North American countries, whose overall crime rates are usually lower in many other spheres [36, 37, 40, 69, 70]. For example, Horii and Burgess’s (2012) [6] reveal that 48.7% of women aged 20 years or more had at least one experience of being harassed in Japan.

Consistently, the studies included in this systematic review tend to endorse the idea that sexual harassment against women is a rather widespread problem that needs to be globally addressed as an actual concern for countries’ safety and security. Indeed, several of the reviewed studies endorse the assumption that women are more susceptible to socially and psychologically unpleasant experiences when they become more spatially mobile and public transport produces almost an inherent perception of insecurity among women. Hence, women are commonly described as implicitly and/or explicitly forced to employ certain preventive strategies to minimize their chances of experiencing harassment [38, 43].

In terms of behavioral adaptations, the most usual ones have shown to be traveling accompanied (even if it makes trips longer or involves route changes), avoiding certain locations, stations, or ’problematic’ transport stops, and refraining from using public transport at night and during certain conflictive schedules [37, 39, 56]. Such strategies are applied in contexts perceived as less safe, such as empty stations, crowded carriages, and poorly lit areas [38].

Furthermore, regarding the conceptual lack of agreement -which dominates attempts to define these practices of harassment-, there is a reliance on binaries; either something is ‘street harassment’ or it is not. Nevertheless, studies such as Vera-Gray (2016) [65] suggest a return to the way such practices on public harassment were conceptualized–in early work on violence against women–as intrusions, reframing street harassment as men’s stranger intrusion. Despite the acknowledgment of the term’s limitations, this may help fill some of the gaps in the existing literature, even further when current literature makes difficult cross-cultural comparisons of, e.g., prevalence rates and harassment patterns across countries due to the ways that the concept is defined [12].

### Policing on harassment: No consensus, no action?

The reviewed studies constantly highlight a contrast between the commonly high governmental awareness of the problem and the scarcity of actions to enhance women’s safety and security in transport [58, 71]. Overall, previous public health and policy responses tend to encourage moving beyond individual-level approaches, considering how structural and interpersonal levels of violence (mediated by typically gendered power relations) are deeply related to harassment outcomes [19].

Sinha et al. (2017) [72] have accounted for the importance of ecological frameworks on policing, including women’s groups with different priorities and ideological positions and evolving social networks among them to combat hegemonic figures and currents operating within the institution of family, state, and civil society, as a first step for raising preventive strategies. Their evidence supports that, for instance, in the Delhi Metro in India, women rate their travel experience more positively compared to other public spaces because of the safety measures put in place [58].

Similarly, other studies covering countries such as Japan [48], Mexico [54, 73], India [74], Brazil [75], Malaysia, Egypt [76], and Nepal [5] show relatively positive appraisals of previous measures in these spaces (e.g., women-only subway cars, buses, and/or cabs, along with social awareness campaigns) to prevent sexual harassment and violence against women. However, other studies state that, although these types of measures can still be potentially effective if correctly conducted, females’ self-reported data tend to consider surveillance cameras and increased police patrols more effective, subsequently linked to a lower degree of fear of crime [47, 48]. This is also coherent with the fact that women’s perception of physical measures, e.g., women-only wagons might be heterogeneous depending on key factors such as the quality of the infrastructure, passenger crowd patterns, and other variables that influence their assessment of the measure in comparison with other preventive actions [48, 54].

Therefore, on a practical level, there can be stated the existence of a certain discrepancy between the measures implemented by governments and entities and the actual women’s perception of them [36]. In this line, there are other experiences that incorporate the view of civil society, feminist associations, and the expertise of departments of "Gender Equality." This is the case of a new program implemented by València City Council called "the violet stops," which incorporates night stops "on request" for female travelers on all their public buses [77, 78].

Coherently, women’s involvement in decision-making has been stressed as a key possibility by Agrawal & Sharman (2015) [79], even though considering harassers’ perspectives may be useful according to other reviewed sources. For instance, there is no official reporting of what happened because the victims’ perception is that it would be futile [40, 41]. Due to this, harassers have been found to feel a low risk of being caught engaging in this behavior [80].

Moreover, it is claimed that using public transportation enables harassers because it gives them anonymity, proximity to victims, and thus the opportunity to partake in harassing behavior [42]. Additionally, studies addressing cultural specificities have stated that the perception of impunity for this and potentially related behaviors can be coupled with men’s acceptance of harassment. Zietz & Das (2018) [81] identified traditional gender norms that support harassment and male sexual entitlement ideologies in India. Thus, many did not comprehend women’s insecurity at the possibility of experiencing harassing situations.

### Harassment co-morbidities: Social determinants and static risk factors

In terms of exploring harassment outcomes among women, literature tends to agree that structural factors can have negative impacts on the social determinants of health and increase the risk of interpersonal violence against women [19]. Transit harassment is, however, stated as gender-specific harm eliciting masked feelings of invasion, anger, humiliation, fear, emotional distress, and moods of disempowerment [82]. Furthermore, a recent meta-analysis conducted by Ranganathan et al. (2021) [12] endorses positive associations between sexual harassment and symptoms of poor mental health, which contributes to its deterioration, and establishes evidence of a significant association between sexual harassment and symptoms of depression.

Demographic factors (e.g., age, education, or city size) have also been shown to have an impact on women’s transportation dynamics [41]. In addition, factors such as perceived safety in the urban environment and being a victim or a witness of a situation of violence or harassment are significant [45], although they depend on the socioeconomic characteristics of the country, as well as the personal situation of the user. In this sense, in developed countries, women, as part of their harassment prevention strategies, are able to select other means of public or private transport to make their routine trips [37].

However, recent studies show that this is not as simple as it sounds, especially in emerging countries. Women, traditionally a particularly vulnerable population group in socio-economic settings, often face economic burdens when, among other issues, they have to shift to a ’safer’ transportation means to avoid potential victimization [83]. This low-income-related ’risk pattern’ implies that, on many occasions, they do not have the option of changing their mode of transport. As a result, they commonly remain more susceptible to vulnerability and face harassment on a daily basis. In other words, gendered inequities in transport are not only evident in the higher levels of harassment experienced by women in these environments but also in the restricted range of behavioral choices they have due to comparatively poorer financial settings [60].

Furthermore, in countries such as Chile, women have been claimed to perform a disproportionately high number of walking movements in urban areas, which also illustrates the inequalities in transport dynamics [52]. Thus, although the ways in which this circumstance is dealt with are diverse, in all cases, there are invisible barriers that prevent women from moving freely and safely around their place of residence. Therefore, explaining to a certain extent behavioral modifications (e.g., changing trip schedules, places, and choices) that, apart from being logically inconvenient, may deepen the already existing inequalities among genders in many aspects [38, 46, 84, 85].

Finally, and in connection to the question opening the heading of this paper, it can be stated that harassment-related dynamics, risks, and *psychosocial traumatisms* commonly present in public transport environments constitute, in words of the existing literature, a widespread and global (although under-acknowledged) problem that, still, represents many functional and structural impacts for women’s daily life, well-being, and travel behavior [57, 73]. Meanwhile, it also increases gender gaps and disparities, which corroborates the necessity for further solutions that may guarantee women’s safety, security, and welfare.

### Limitations of the study and further research

A systematic review, despite being a valuable research tool, may encounter various limitations. One of the primary concerns is publication bias, where studies with positive results are more likely to be published, while those with negative results may not be as visible, potentially distorting the overall picture of available evidence [86]. Additionally, the exclusion of studies in languages other than English and Spanish may introduce a language bias, limiting the representativeness of the sample and potentially excluding relevant evidence available in other languages [87].

Another potential limitation lies in selection bias, as some studies may not have been identified during the search, leading to an incomplete representation of the existing literature [88]. There is also the possibility of variability in the methodological quality of the studies included in the review, which could impact the identified findings. However, these limitations have been minimized by strictly following PRISMA guidelines, ensuring a transparent, systematic process with clear inclusion/exclusion criteria. Additionally, the Critical Appraisal Skills Programme has been applied to certify that the selected articles meet sufficient quality requirements [63].

Moreover, a substantial shortage of articles addressing the situation of transgender women in public transportation has been identified. This underscores the need for broader and more specific research on their experiences and particular challenges in this context. The lack of attention in the literature emphasizes the importance of addressing the diversity of gender experiences in future research, as well as in actions related to the planning and evaluation of public transportation policies, ensuring that the needs and concerns of all individuals are recognized and appropriately addressed.

## Conclusions

The results of this systematic review, which addressed the far-reaching problem of violence and harassment against women in transit environments in literature, and distinctively exercised in public transportation, allow us to raise some key conclusions:

Firstly, despite a limited body of research, its extensive series of impacts on women’s safety, security, inclusion, health, and welfare is consistently endorsed in the literature.

Secondly, apart from being largely explained by existing inequalities grounded on social determinants, transit harassment also deepens gender gaps further, acquiring prospective importance for transport settings.

Finally, and although their scope does not usually reach harassment policing, most studies highlight the need for systematic actions beyond perceptions, enhancing opportunities for further improvements in health, lifestyle, and overall well-being among female travelers using public transportation worldwide.

## Supporting information

S1 Checklist PRISMA checklist for systematic reviews. (ZIP)

## References

[1] Basagaña X., Triguero-Mas M., Agis D., Pérez N., Reche C., Alastuey A., et al. (2018). Effect of public transport strikes on air pollution levels in Barcelona (Spain). *Science of the total environment*, 610: 1076–1082. doi: 10.1016/j.scitotenv.2017.07.263 28847101

[2] Lawrence R. J. (2019). Human ecology in the context of urbanization. In: *Integrating Human Health into Urban and Transport Planning* (pp. 89–109). Springer, Cham. 10.1007/978-3-319-74983-9_6

[3] Useche S.A., Marin C., & Llamazares F. (2023). “Another (hard) day moving in the city”: Development and validation of the MCSS, a Multimodal Commuting Stress Scale. *Transportation Research Part F*: *Traffic Psychology and Behaviour* [Paper Under Evaluation].

[4] Ball K. S., & Wesson C. J. (2017). Perceptions of unwanted sexual behaviour on public transport: exploring transport density and behaviour severity. *Crime prevention and community safety*, 19, 199–210. 10.1057/s41300-017-0026-3

[5] Gautam N., Sapakota N., Shrestha S., & Regmi D. (2019). Sexual harassment in public transportation among female student in Kathmandu valley. *Risk Management and Healthcare Policy*, 12, 105–113. doi: 10.2147/RMHP.S196230 31360074 PMC6625743

[6] Horii M., & Burgess A. (2012). Constructing sexual risk: “Chikan”, collapsing male authority and the emergence of women-only train carriages in Japan. *Health Risk & Society*, 14, 41–55. 10.1080/13698575.2011.641523

[7] Loukaitou-Sideris A. (2014). Fear and safety in transit environments from the women’s perspective. *Security journal* 27(2): 242–256. 10.1057/sj.2014.9

[8] Hoor-Ul-Ain S. (2020). Public sexual harassment mayhem on public transport in megacities-Karachi and London: A comparative review. *Aggression and Violent Behavior*, 52, 101420. 10.1016/j.avb.2020.101420

[9] Crouch M. (2009). Sexual harassment in public places. *Social Philosophy Today*, 25, 137–148. 10.5840/socphiltoday20092511

[10] Vera-Gray F., & Kelly L. (2020). Contested gendered space: public sexual harassment and women’s safety work. *International Journal of Comparative and Applied Criminal Justice*, 44(4), 265–275. 10.1080/01924036.2020.1732435

[11] Ceccato V., & Uittenbogaard A. C. (2014). Space–time dynamics of crime in transport nodes. *Annals of the Association of American geographers*, 104(1), 131–150. 10.1080/00045608.2013.846150

[12] Ranganathan M., Wamoyi J., Pearson I., & Stöckl H. (2021). Measurement and prevalence of sexual harassment in low-and middle-income countries: a systematic review and meta-analysis. *BMJ Open*, 11(6), e047473. doi: 10.1136/bmjopen-2020-047473 34168030 PMC8231049

[13] Spitzberg B.H. (2017). Acknowledgment of unwanted pursuit, threats, assault, and stalking in a college population. *Psychological Violence*, 7(2), 265–275.

[14] DelGreco M., Ebesu Hubbard A.S. & Denes A. (2021). Communicating by catcalling: Power dynamics and communicative motivations in street harassment. *Violence Against Women*, 27(9), 1402–1426. doi: 10.1177/1077801220927085 32567540

[15] Ceccato V., & Loukaitou-Sideris A. (Eds.) (2020). *Transit crime and sexual violence in cities*: *International evidence and prevention*. Routledge.

[16] Gardner N., Cui J., & Coiacetto E. (2017). Harassment on public transport and its impacts on women’s travel behaviour. *Australian Planner*, 54(1), 8–15. 10.1080/07293682.2017.1299189

[17] Ceccato V., Näsman P., & Langefors L. (2020). Sexual violence on the move: An assessment of Youth’s victimization in public transportation. *Women & Criminal Justice*, 31(4), 294–312. 10.1080/08974454.2020.1733732

[18] Thurston W.E., & Vissandjee B. (2005). An ecological model for understanding culture as a determinant of women’s health. *Critical Public Health*, 15(3), 229–242. 10.1080/09581590500372121

[19] Montesanti S. R., & Thurston W. E. (2015). Mapping the role of structural and interpersonal violence in the lives of women: implications for public health interventions and policy. *BMC Womens Health*, 11(15), 100. doi: 10.1186/s12905-015-0256-4 26554358 PMC4641364

[20] Lea S. G., D’Silva E., & Asok A. (2017). Women’s strategies addressing sexual harassment and assault on public buses: an analysis of crowdsourced data. *Crime Prevention and Community Safety*, 19(3), 227–239. 10.1057/s41300-017-0028-1

[21] Mohamed A. A., & Stanek D. (2020). The influence of street network configuration on sexual harassment patterns in Cairo. *Cities*, 98, 102583. 10.1016/j.cities.2019.102583

[22] Abenoza R. F., Ceccato V., Susilo Y. O., & Cats O. (2018). Individual, travel, and bus stop characteristics influencing travelers’ safety perceptions. *Transportation Research Record*, 2672(8), 19–28. 10.1177/0361198118758677

[23] Women UN. (2019). *Addressing violence and harassment against women in the world of work*. New York: United Nations. Available at: https://www.unwomen.org/-/media/headquarters/attachments/sections/library/publications/2019/addressing-violence-and-harassment-against-women-in-the-world-of-work-en.pdf?la=en&vs=4050

[24] Rader N. E., & Haynes S. H. (2011). Gendered fear of crime socialization: An extension of Akers’s social learning theory. *Feminist Criminology*, 6(4), 291–307

[25] Fileborn B., & O’Neill T. (2021). From “Ghettoization” to a Field of Its Own: A Comprehensive Review of Street Harassment Research. *Trauma*, *Violence*, *& Abuse*, 24(1), 125–138. 10.1177/15248380211021608 34098825

[26] Almanza-Avendaño A. M., Romero-Mendoza M., & Gómez A. H. (2022). From harassment to disappearance: Young women’s feelings of insecurity in public spaces. *PLoS ONE*, 17(9), e0272933. doi: 10.1371/journal.pone.0272933 36070257 PMC9451059

[27] BTP—British Transport Police. (2015.) Report it to stop it: Tackling unwanted sexual behaviour on public transport. Available at: http://www.btp.police.uk/advice_and_information/how_we_tackle_crime/report_it_to_stop_it.aspx

[28] TfL—Transport for London. (2016). ‘Report it to stop it’ increases public confidence to report unwanted sexual behaviour. [online]. https://tfl.gov.uk/info-for/media/press-releases/2016/february/-report-it-to-stop-it-increases-public-confidence-to-report-unwanted-sexual-behaviour

[29] Stringer S.M. (2007). Hidden in plain sight: Sexual harassment and assault in the New York City transport system. New York: Manhattan Borough President Office.

[30] Claudia Y. M., & Rahaditya R. (2020). Legal Protection Against Female Victims of Sexual Abuse on Commuter Line Rangkas Bitung–Tanah Abang Route. In *The 2nd Tarumanagara International Conference on the Applications of Social Sciences and Humanities (TICASH 2020)* (pp. 937–941). Atlantis Press. 10.2991/assehr.k.201209.147

[31] Yañez-Pagans P., Martinez D., Mitnik O. A., Scholl L., & Vazquez A. (2019). Urban transport systems in Latin America and the Caribbean: lessons and challenges. *Latin American Economic Review*, 28(1), 1–25. 10.1186/s40503-019-0079-z

[32] Levy C. (2013). Travel choice reframed: “deep distribution” and gender in urban transport. *Environment and* Urbanization, 25(1), 47–63. 10.1177/0956247813477810

[33] Armstrong R., Hall B. J., Doyle J., & Waters E. (2011). ‘Scoping the scope’of a cochrane review. *Journal of Public Health*, 33(1), 147–150. 10.1093/pubmed/fdr015 21345890

[34] Arksey H., & O’Malley L. (2005). Scoping studies: towards a methodological framework. *International Journal of Social Research Methodology*, 8(1), 19–32. 10.1080/1364557032000119616

[35] Moher D., Altman D. G., Liberati A., & Tetzlaff J. (2011). PRISMA statement. *Epidemiology*, 22(1), 128. doi: 10.1097/EDE.0b013e3181fe7825 21150360

[36] Loukaitou-Sideris A., & Fink C. (2009). Addressing women’s fear of victimization in transportation settings: A survey of US transit agencies. *Urban Affairs Review*, 44(4), 554–587. 10.1177/1078087408322874

[37] Ceccato V., Langefors L., & Näsman P. (2021). The impact of fear on young people’s mobility. *European Journal of Criminology*, 2021. doi: 10.1177/14773708211013299

[38] Orozco-Fontalvo M., Soto J., Arévalo A., & Oviedo-Trespalacios O. (2019). Women’s perceived risk of sexual harassment in a Bus Rapid Transit (BRT) system: The case of Barranquilla, Colombia. *Journal of Transport & Health*, 14, 100598. 10.1016/j.jth.2019.100598

[39] Mishra D., & Lamichhane J. (2018). Experience of sexual harassment in public transport among female health science students: A cross sectional study of Kathmandu, Nepal. *Journal of Manmohan Memorial Institute of Health Sciences*, 4(1), 20–32. 10.3126/jmmihs.v4i1.21134

[40] Lewis S., Saukko P., & Lumsden K. (2021). Rhythms, sociabilities and transience of sexual harassment in transport: Mobilities perspectives of the London underground. *Gender*, *Place & Culture*, 28(2), 277–298. 10.1080/0966369X.2020.1734540

[41] Quinones L. M. (2020). Sexual harassment in public transport in Bogotá. *Transportation Research Part A*: *Policy and Practice*, 139, 54–69. 10.1016/j.tra.2020.06.018

[42] Neupane G., & Chesney-Lind M. (2014). Violence against women on public transport in Nepal: Sexual harassment and the spatial expression of male privilege. *International Journal of Comparative and Applied Criminal Justice*, 38(1), 23–38. 10.1080/01924036.2013.794556

[43] Tripathi K., Borrion H., & Belur J. (2017). Sexual harassment of students on public transport: An exploratory study in Lucknow, India. *Crime prevention and community safety*, 19(3), 240–250. 10.1057/s41300-017-0029-0

[44] Madan M., & Nalla M. K. (2016). Sexual harassment in public spaces: Examining gender differences in perceived seriousness and victimization. *International Criminal Justice Review*, 26(2), 80–97. 10.1177/1057567716639093

[45] Alonso F. M., Useche S. A., Faus M., & Esteban C. (2020). Does urban security modulate transportation choices and travel behavior of citizens? A national study in the Dominican Republic. *Frontiers in Sustainable Cities*, 2, 42. 10.3389/frsc.2020.00042

[46] Infante-Vargas D., & Boyer K. (2021). Gender-based violence against women users of public transport in Saltillo, Coahuila, Mexico. *Journal of Gender Studies*, 31(2), 216–230. 10.1080/09589236.2021.1915753

[47] Coppola P., & Silvestri F. (2020). Assessing travelers’ safety and security perception in railway stations. *Case Studies on Transport Policy*, 8(4), 1127–1136. 10.1016/j.cstp.2020.05.006

[48] Shibata S. (2020). Are women-only cars (WOC) a solution to groping? A survey among college students in Tokyo/Kanagawa, Japan. *International Journal of Comparative and Applied Criminal Justice*, 44(4), 293–305. 10.1080/01924036.2020.1719533

[49] Soto J., Orozco-Fontalvo M., & Useche S. A. (2022). Public transportation and fear of crime at BRT Systems: Approaching to the case of Barranquilla (Colombia) through integrated choice and latent variable models. *Transportation Research Part A*: *Policy and Practice*, 155, 142–160. 10.1016/j.tra.2021.11.001

[50] Vasquez-Henriquez P., Graells-Garrido E., & Caro D. (2020). Tweets on the Go: Gender Differences in Transport Perception and Its Discussion on Social Media. *Sustainability*, 12(13): 5405. 10.3390/su12135405

[51] Ceccato V., & Loukaitou-Sideris A. (2022). Fear of sexual harassment and its impact on safety perceptions in transit environments: a global perspective. *Violence Against Women*, 28(1), 26–48. doi: 10.1177/1077801221992874 33656953 PMC8564253

[52] Sagaris L., & Tiznado-Aitken I. (2020). Sustainable Transport and Gender Equity: Insights from Santiago, Chile. In: Oviedo D., Duarte N.V., & Pinto A. M. A. (Eds.). *Urban Mobility and Social Equity in Latin America*: *Evidence*, *Concepts*, *Methods* (*Transport and Sustainability*, *Vol*. 12), Emerald Publishing Limited, Bingley, 103–134. 10.1108/S2044-994120200000012009

[53] Lebugle A. (2017). Young women in large cities are the main victims of violence in public space. *Population Societies*, 11, 1–4. 10.3917/popsoc.550.0001

[54] Dunckel-Graglia A. (2016). Finding mobility: Women negotiating fear and violence in Mexico City’s public transit system. *Gender*, *Place & Culture*, 23(5), 624–640. 10.1080/0966369X.2015.1034240

[55] Wa M., & Samper D.A. (2006). "No mercy, no remorse": personal experience narratives about public passenger transportation in Nairobi, Kenya. *Africa Today*, 51–81. Available at: https://www.jstor.org/stable/4187721?seq=1#metadata_info_tab_contents

[56] Lynch G., & Atkins S. (1988). The influence of personal security fears on women’s travel patterns. *Transportation*, 15(3), 257–277. 10.1007/BF00837584

[57] Reed E., Salazar M., Agah N., Behar A. I., Silverman J. G., Walsh-Buhi E., et al. (2019). Experiencing sexual harassment by males and associated substance use & poor mental health outcomes among adolescent girls in the US. *SSM-Population Health*, 9, 100476. 10.1016/j.ssmph.2019.100476 31998825 PMC6978506

[58] Gopal K., & Shin E. J. (2019). The impacts of rail transit on the lives and travel experiences of women in the developing world: Evidence from the Delhi Metro. *Cities*, 88, 66–75. 10.1016/j.cities.2019.01.008

[59] Vanderschuren M. J., Phayane S. R., & Gwynne-Evans A. J. (2019). Perceptions of gender, mobility, and personal safety: South Africa moving forward. *Transportation Research Record*, 2673(11): 616–627. 10.1177/0361198119854087

[60] Medina G., & Zapana A. E. (2016). Representaciones sociales de las mujeres jóvenes sobre el acoso sexual callejero en la ciudad de Puno. *Punto Cero*, 21(33), 61–84.

[61] Ison J., Forsdike K., Henry N., Hooker L., & Taft A. (2023). “You’re just constantly on alert”: Women and Gender-Diverse People’s Experiences of Sexual Violence on Public Transport. *Journal of interpersonal violence*, 38(21–22), 11617–11641. doi: 10.1177/08862605231186123 37465905 PMC10515455

[62] Lee J., Mao R., & Pervez A. (2024). Perceived risk of crime on driverless public bus and ride-pooling services in China. Travel Behaviour and Society, 35, 100730. 10.1016/j.tbs.2023.100730

[63] Long H. A., French D. P., & Brooks J. M. Optimising the value of the critical appraisal skills programme (CASP) tool for quality appraisal in qualitative evidence synthesis. Research Methods in Medicine & Health Sciences (2020) 1:1:31–42. doi: 10.1177/2632084320947559

[64] Tran M. (2015). Combatting gender privilege and recognizing a woman’s right to privacy in public spaces: Arguments to criminalize catcalling and creepshots. *Hastings Women’s Law Journal*, 26, 185–206. Available at: https://repository.uchastings.edu/hwlj/vol26/iss2/1

[65] Vera-Gray F. (2016). Men’s stranger intrusions: Rethinking street harassment. *Women’s Studies International Forum*, 58, 9–17. 10.1016/j.wsif.2016.04.001

[66] Natarajan M., Schmuhl M., Sudula S., & Mandala M. (2017). Sexual victimization of college students in public transport environments: A whole journey approach. *Crime Prevention and Community Safety*, 19(3), 168–182. 10.1057/s41300-017-0025-4

[67] Chockalingam K., & Vijaya A. (2008). Sexual harassment of women in public transport in Chennai city. A Victimological Perspective. *The Indian Journal of Criminology and Criminalists*, 29(3), 167–184.

[68] Kirchhoff G. F., Morosawa H., Barkhuizen J., Bussinger C., Sutseyo H., & Bey J. F. (2007). The Asian passengers’ safety study of sexual molestation on trains and buses: The Indonesian pilot study. *Acta Criminologica*, 20, 1–13.

[69] Macassa G., Winersjö R., Wijk K., McGrath C., Ahmadi N., & Soares J. (2018). Fear of crime and its relationship to self-reported health and stress among men. *Journal of Public Health Research*, 6(3), 1010. doi: 10.4081/jphr.2017.1010 29441331 PMC5806035

[70] Fyhri A., & Backer-Grøndahl A. (2012). Personality and risk perception in transport. *Accident Analysis & Prevention*, 49, 470–475. doi: 10.1016/j.aap.2012.03.017 23036425

[71] Smith M. J. (2008). Addressing the security needs of women passengers on public transport. *Security Journal*, 21(1), 117–133. 10.1057/palgrave.sj.8350071

[72] Sinha P., Gupta U., Singh J., & Srivastava A. (2017). Structural violence on women: An impediment to women empowerment. *Indian journal of community medicine*: *official publication of Indian Association of Preventive & Social Medicine*, 42(3), 134. doi: 10.4103/ijcm.IJCM_276_15 28852274 PMC5561688

[73] Dunckel-Graglia A. (2013). Women-only transportation: How “pink” public transportation changes public perception of women’s mobility. *Journal of Public Transportation*, 16(2), 5. 10.5038/2375-0901.16.2.5

[74] Tara S. (2011). Private space in public transport: Locating gender in the Delhi metro. *Economic and Political Weekly*, 71–74. Available online: https://www.jstor.org/stable/23065551

[75] Tillous M. (2020). Women, (railway) class and the state: an analysis of two controversies surrounding women-only metro carriages (Cairo–São Paulo). *Gender*, *Place & Culture*, 27(8), 1155–1175. 10.1080/0966369X.2019.1654435

[76] García V. M. S. (2019). Misoginia en el espacio público, femicidio no íntimo y prueba criminal. *Estado & comunes*, *revista de políticas y problemas públicos*, 1(8).

[77] EMT Valencia (2021). [EMT reactivates the ’violet stops’ pilot program on night lines]. Available at: https://emtvalencia.info/es/2021/03/emt-reactiva-el-programa-piloto-de-las-paradas-violeta-en-las-lineas-nocturnas/

[78] Ceccato V., & Paz Y. (2017). Crime in São Paulo’s metro system: Sexual crimes against women. *Crime Prevention and Community Safety*, 19(3), 211–226. 10.1057/s41300-017-0027-2

[79] Agrawal A., & Sharma A. (2015). Gender contests in the Delhi Metro: Implications of reservation of a coach for women. *Indian Journal of Gender Studies*, 22(3), 421–436. 10.1177/0971521515594279

[80] Rounsevell H. R. (2015). Derechos en conflicto: una ley anti-piropo en España. *Cuestiones de género*: *de la igualdad y la diferencia*, 10, 151–160.

[81] Zietz S., & Das M. (2018). ‘Nobody teases good girls’: A qualitative study on perceptions of sexual harassment among young men in a slum of Mumbai. *Global Public Health*, 13(9), 1229–1240. doi: 10.1080/17441692.2017.1335337 28580845 PMC6690339

[82] Pearson A. L., & Breetzke G. D. (2013). The association between the fear of crime, and mental and physical wellbeing in New Zealand. *Social Indicators Research*, 119(1), 281–294. 10.1007/s11205-013-0489-2

[83] Shabbir M. S., Abbas M., Aman Q., Ali R., & Orangzeb K. (2019). Poverty Reduction Strategies. Exploring the link between Poverty and Corruption from less developed countries. *Dilemas Contemporáneos*: *Educación*, *Política y Valores* 6(2), 49.

[84] Bailey B. (2017). Greetings and compliments or street harassment? Competing evaluations of street remarks in a recorded collection. *Discourse & Society*, 28(4), 353–373. 10.1177/0957926517702979

[85] Bastomski S. & Smith P. (2017). Gender, fear, and public places: How negative encounters with stranger’s harm women. *Sex Roles*, 76(1–2), 73–88. 10.1007/s11199-016-0654-6

[86] Dwan K., Gamble C., Williamson P. R., Kirkham J. J., & Reporting Bias Group. (2013). Systematic review of the empirical evidence of study publication bias and outcome reporting bias—an updated review. *PloS one*, 8(7), e66844. doi: 10.1371/journal.pone.0066844 23861749 PMC3702538

[87] Grzybowski A., & Kanclerz P. (2019). Language bias and methodological issues in determining reliable evidence for systematic reviews. *JAMA ophthalmology*, 137(1), 118–119. doi: 10.1001/jamaophthalmol.2018.4945 30347027

[88] Williamson P. R., & Gamble C. (2005). Identification and impact of outcome selection bias in meta‐analysis. *Statistics in medicine*, 24(10), 1547–1561. doi: 10.1002/sim.2025 15580591

